# MRI of the Achilles tendon – a comprehensive pictorial review. Part two

**DOI:** 10.1016/j.ejro.2021.100343

**Published:** 2021-03-27

**Authors:** Pawel Szaro, Katarina Nilsson-Helander, Michael Carmont

**Affiliations:** a Department of Radiology, Institute of Clinical Sciences, Sahlgrenska Academy, University of Gothenburg, Gothenburg, Sweden; b Department of Musculoskeletal Radiology, Sahlgrenska University Hospital, Gothenburg, Sweden; c Department of Descriptive and Clinical Anatomy, Medical University of Warsaw, Warsaw, Poland; d Department of Orthopedics, Institute of Clinical Sciences, Sahlgrenska Academy, University of Gothenburg, Sweden; e The Department of Orthopaedic Surgery, Princess Royal Hospital, Shrewsbury & Telford Hospital NHS Trust, Shropshire, UK

**Keywords:** Achilles tendon, Tendinopathy, Tendinosis, Haglund’s syndrome, Rupture

## Abstract

The most common disorder affecting the Achilles tendon is midportion tendinopathy. A focal fluid signal indicates microtears, which may progress to partial and complete rupture. Assessment of Achilles tendon healing should be based on tendon morphology and tension rather than structural signal. After nonoperative management or surgical repair of the Achilles tendon, areas of fluid signal is pathologic because it indicates re-rupture. A higher signal in the postoperative Achilles tendon is a common finding and is present for a prolonged period following surgical intervention and needs to be interpreted alongside the clinical appearance.

## Highlights

• The abnormalities on MRI should be correlated with the clinical findings.
• Higher signal of the Achilles tendon is a common postoperative finding.
• The fluid signal within the Achilles tendon graft indicates a rupture.
• Postoperative complications of Haglund’s syndrome should be assessed on MRI.
• Elongation of the Achilles tendon is seen after surgical or conservative treatment.

## 1 Paratenonitis

**Fig. 1:**
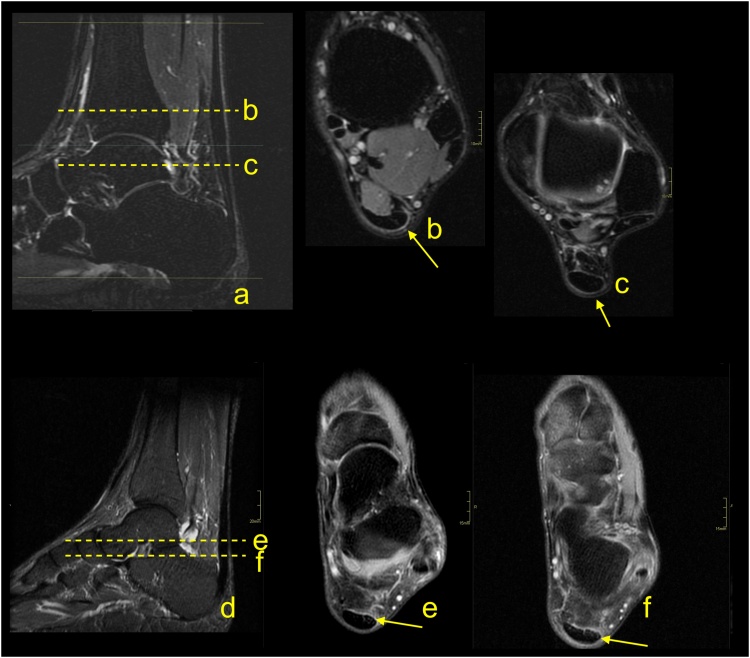
Paratenonitis. Two patients with pain of the Achilles tendon. a–c: a 32-year-old patient with the pain of the Achilles tendon after running. d–f: a 56-year-old patient with pain of the Achilles tendon without training. MRI (T2-weighted with fat suppression) revealed a higher signal of the paratenon (b, c, e and f) with insignificant tendinopathy of the Achilles tendon (a–c).

Paratenonitis is an inflammatory reaction of the paratenon, which may be compared to tenosynovitis and tenovaginitis in tendons with a synovial sheath [1]. Paratenonitis may accompany Achilles tendon tendinopathy or be a separate pathology (Fig. 1).

In trauma or overuse conditions (e.g., midportion or insertional tendinopathy), the signal of the paratenon is higher, which is probably due to higher vascularization and edema [2]. The swelling, which is associated with inflammation, reduces the smooth gliding of the tendon within the nerve rich paratenon, stimulating pain. Edema may also be seen in the adjacent part Kager's fat pad and the paratenon (Fig. 1) [3]. The higher signal in the paratenon may be seen in some patients with systemic inflammatory diseases [4]. In systemic hyperuricemia, uric acid crystals may be deposited within the tendon, but more commonly, they are deposited in the lateral part of the Achilles paratenon [5], which may lead to focal thickening mimicking localized paratenonitis [6].

## 2 Tendinopathy

### 2.1 Midportion tendinopathy

**Fig. 2:**
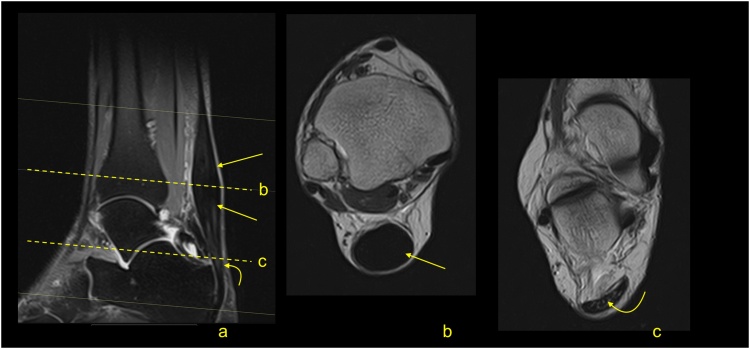
Midportion tendinopathy of the Achilles tendon. The MRI (a–c) revealed the thickening of the Achilles tendon (straight arrow) in the midportion, while the insertion is regular (curved arrow). A- T2-weighted with fat suppression, b and c- PD-weighted.

**Fig. 3:**
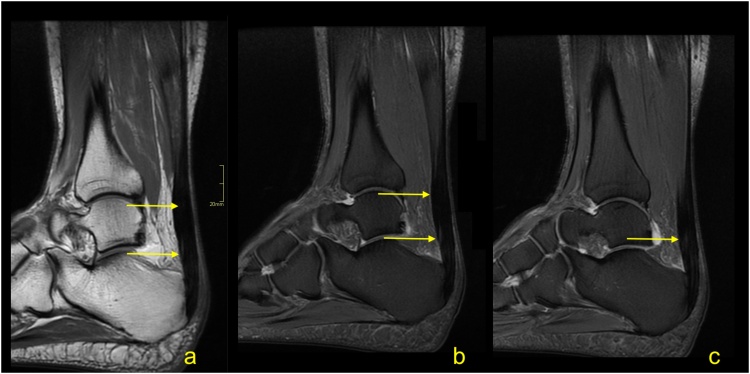
Tendinopathy of the whole Achilles tendon (arrow) in a 62-year-old patient with pain for more than 5 years. The MRI revealed thickening of the whole Achilles tendon (arrow). A- PD-weighted, b and c- PD-weighted with fat suppression.

Midportion Achilles tendinopathy is a common problem, affecting 30 % of the general population and as many as 50 % of athletes [7,8]. The most common cause is training overload causing microtrauma. The common sign of tendinosis is fusiform thickening of the midportion of the Achilles tendon with increased signal [9] (Fig. 2). Thickening of the whole Achilles tendon rarely occurs (Fig. 3).

**Fig. 4:**
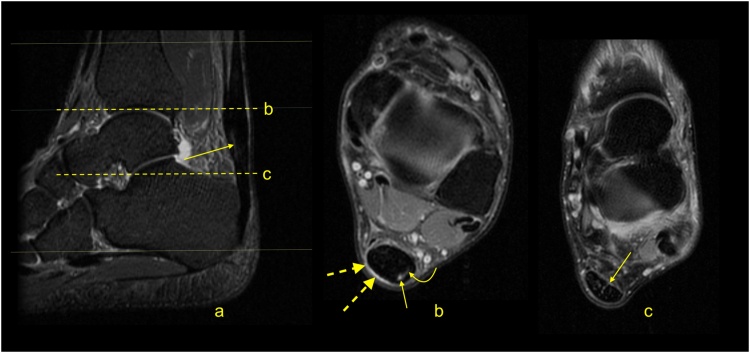
Tendinopathy of the Achilles tendon. A 49-year-old patient with pain of the Achilles tendon. Normal septae between the subtendons are visible (straight arrow). The focal oval higher signal (curved arrow) is visible in the midportion in the subtendon from the medial head of the gastrocnemius. A-STIR, b and c – PD-weighted with fat suppression.

**Fig. 5:**
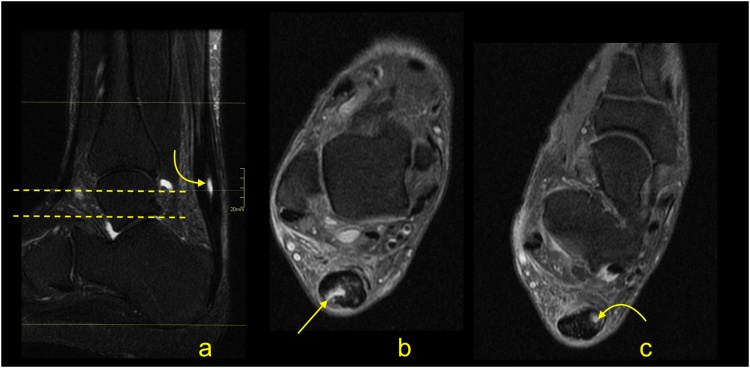
Achilles tendinopathy with intrasubstance rupture (curved arrow) and rupture on the tendon's surface (straight arrow). A 61-year-old patient with pain of the Achilles tendon for many years. A-T2-weighted with fat suppression, b and c- PD-weighted with fat suppression.

With tendinosis, the thickening of the tendon progresses. First, the septae are thicker, then larger areas of higher signal appear, which respond to the internal regeneration process [10]. The external tendon regeneration is often a parallel process and is related to the highly vascularized paratenon. MRI usually reveals a thickened paratenon with circumference of the higher signal around the Achilles tendon (Fig. 4). If the tendinopathy progresses, intrasubstance or surface ruptures may occur (Fig. 5). A complete rupture of the Achilles tendon may be the end-stage of degeneration [11]. Common to the pathophysiological background of tendinopathy are hypoxia and mucoid degeneration. Hypoxic tendinopathy usually presents as a painful thickening in the midportion of the Achilles tendon, which is known to be a vascular watershed [11]. Mucoid degeneration, in contrast to ischemic degeneration, is often clinically asymptomatic [12]. A distinction between mucoid and ischemic degeneration based on classical MRI sequences is challenging; however, it is somewhat possible using new MRI techniques.

**Fig. 6:**
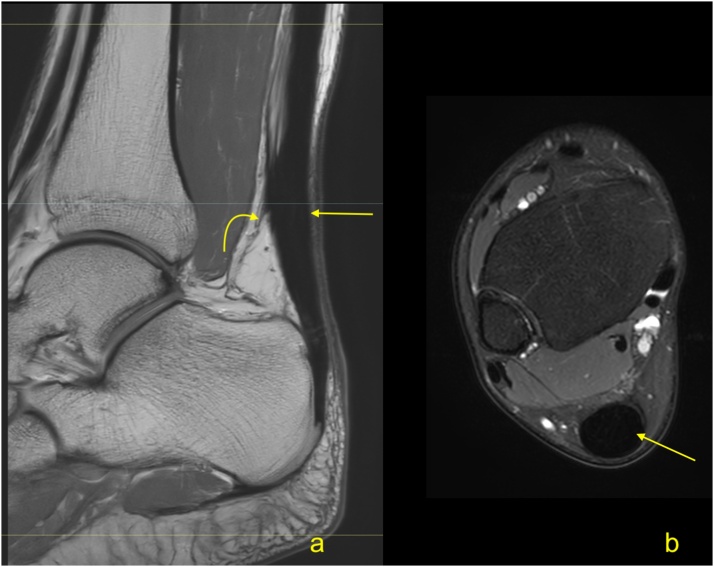
A 63-year-old patient who underwent a partial Achilles tendon rupture about 8 months ago, with an excellent clinical outcome. Remodeling of the Achilles tendon (straight arrow) and adhesions (curved arrow) are visible on MRI. A- PD-weighted, b- PD-weighted with fat suppression.

**Fig. 7:**
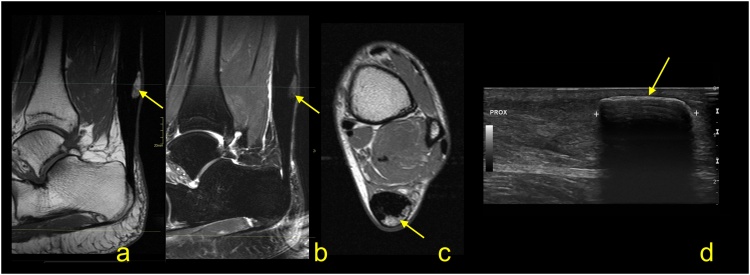
Achilles tendinopathy after many years. More pain after walking in the last 2 months. The MRI (a and b) and ultrasound (c) revealed the presence of superficial calcification (arrow) within a thickened Achilles tendon. A – PD-weighted, b- T2-weighted with fat suppression, c- PD-weighted, d- ultrasound, longitudinal scan of the Achilles tendon.

**Fig. 8:**
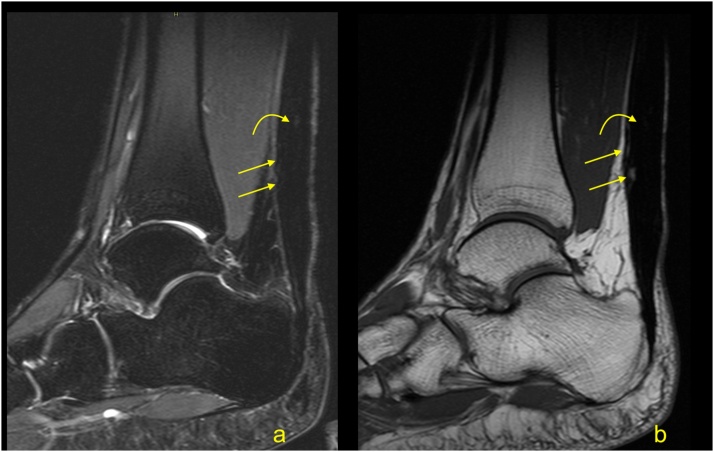
Degeneration of the Achilles tendon. MRI revealed focal fat degeneration (straight arrow), which on T1-weighted imaging shows a higher signal and is suppressed on T2-weighted imaging with fat suppression. There are also the focal fluid lesions (curved arrow). A-T2-weighted with fat suppression, b- T1-weighted.

The progress of degeneration corresponds to more heterogenous areas of higher signal within the Achilles tendon (Fig. 4). During the months and years after injury, the signal of the tendon decreases but thickening usually remains (Fig. 6). Healing of the focal defects of the tendon may be seen as the presence of a diffuse or focal higher signal, even small areas with fluid signal or calcifications (Fig. 7) may appear [13]. Deposition of the intratendinous fat (Fig. 8) or calcification (Fig. 7) correspond to respective fat or bone metaplasia of the Achilles tendon after the trauma of degeneration [14].

**Fig. 9:**
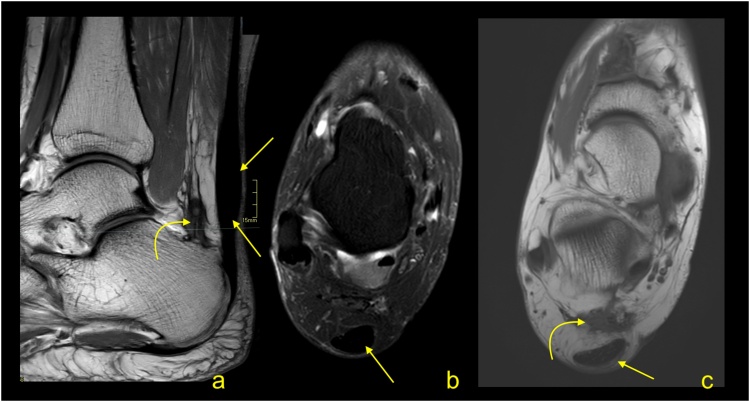
Achilles tendinopathy (straight arrow) was treated five times with sclerosing therapy; fibrotic lesions are seen in Kager's fat pad (curved arrow) at the level of injection. A- PD-weighted, b- PD- weighted with fat suppression, c- PD-weighted.

The treatment of midportion tendinopathy with sclerosing therapy may lead to the formation of fibrotic lesions within Kager's fat pad at the level of injection (Fig. 9).

Tendinopathy is a clinical term regarding tendinitis or tendonitis, consisting of pain, tendon thickening and reduced function [15]. There is some controversy in the nomenclature as some authors recommend the use of the terms "tendinosis" and "tendinitis” or “tendonitis" only after histopathological examination, while the term tendinopathy is a more general term [10]. Although many terms are used in the literature, we have used the term tendinopathy as this is the term used predominately in the current radiological literature.

### 2.2 Insertional tendinopathy

There is some disagreement in the terminology of lesions in the distal third of the Achilles tendon. Some authors use the term insertional tendinopathy, but insertional tendinitis or tendinosis can also be found.

This condition may have two origins, inflammatory or non-inflammatory. Inflammatory tendinopathy is discussed in the section regarding arthritis in part one, whereas non-inflammatory tendinopathy is discussed here. Overuse, mechanical factors and peripheral spondyloarthropathies are common causes of insertional tendinopathy.

**Fig. 10:**
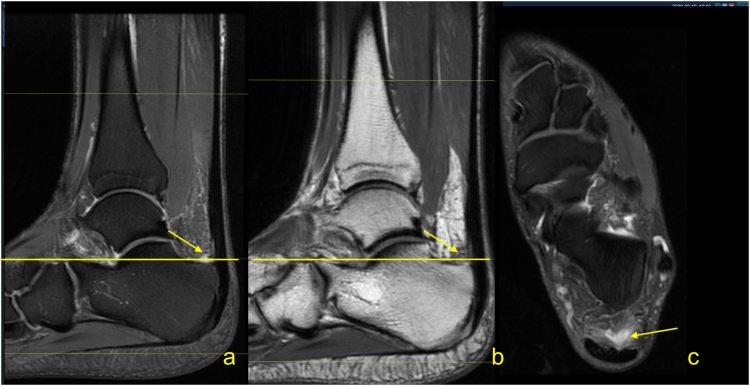
The early stage of Haglund's syndrome. A 32-year-old patient with pain at the insertion of the Achilles tendon with clinical suspicion for Haglund's syndrome. Only effusion and synovitis are visible in the retrocalcaneal bursa (arrow). A-T2- weighted, b- PD- weighted, c-PD- weighted with fat suppression.

**Fig. 11:**
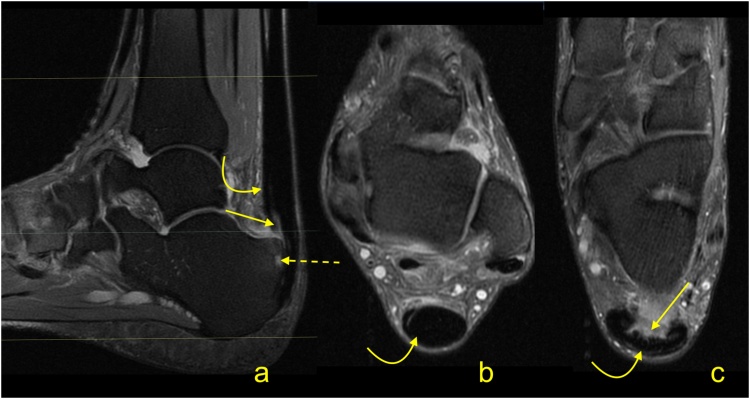
Advanced stage of Haglund's syndrome with partial rupture of the Achilles tendon of subtendons from the soleus and lateral head of the gastrocnemius (straight arrow). The calcaneus is present as a small intraosseous cyst (dashed arrow) surrounded with bone marrow edema. The midportion and insertion of the Achilles tendon are thickened (curved arrow). A-c- PD- weighted with fat suppression.

**Fig. 12:**
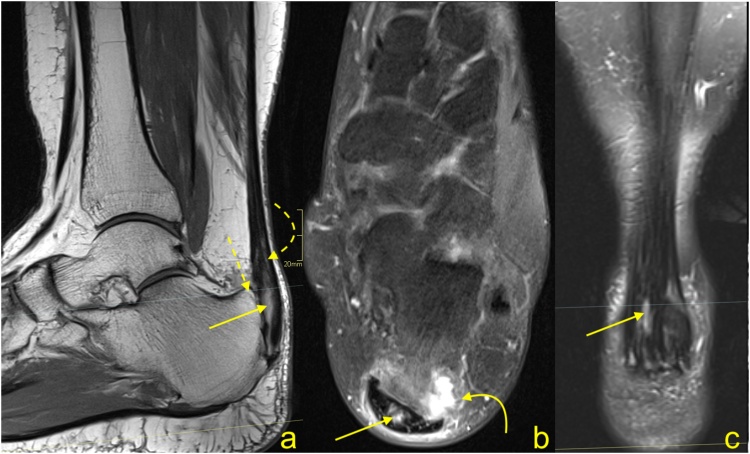
Advanced stage of Haglund's syndrome. A 67-year-old patient with pain at the insertion of the Achilles tendon for more than 5 years. MRI revealed Haglund’s deformity (dashed arrow); alteration in the signal at the insertion, which corresponds to an intrasubstance defect and discrete diffuse calcifications (straight arrow); effusion; and synovitis in the retrocalcaneal bursa (curved arrow). A-PD- weighted, b-PD- weighted with fat suppression, c-T2- weighted with fat suppression.

**Fig. 13:**
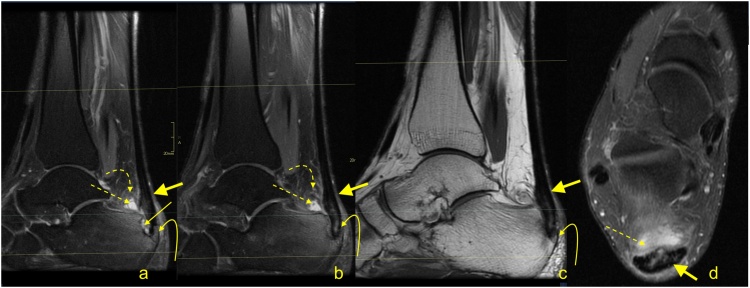
Advanced stage of Haglund's syndrome. MRI revealed synovitis in the retrocalcaneal bursa (dashed arrow) with edema in Kager's fat pad (curved arrow). In the calcaneus intraosseous cyst (straight arrow) with extensive bone marrow edema is visible. The enthesophyte with bone marrow edema is visible in the superficial part of the tendon's insertion (curved arrow). The Achilles tendon in the distal 1/3 shows thickening and alteration in signal (thick arrow). A and b- PD- weighted with fat suppression, c- PD- weighted, d- PD-weighted with fat suppression.

Insertional tendinopathy is a common cause of heel pain and operative treatment is more often indicated than in midportion tendinopathy. If the bony prominence on the posterosuperior part of the calcaneus causes impingement of the Achilles tendon, the condition is called Haglund’s syndrome. The MRI manifestations of the Haglund’s syndrome may initially be subtle, such as retrocalcaneal bursitis, and only small signal changes within the Achilles tendon insertion are seen (Fig. 10). If the pathological process continues, irregular edema and thinning of the Achilles tendon enthesis, thickening of the rest of the distal third of the tendon [16] (Fig. 11) or tendon partial tear may occur (Fig. 12). Bone marrow edema in the adjacent part of the calcaneus [17] and retrocalcaneal bursitis with some superficial bony irregularities are usually seen (Fig. 11, Fig. 13). In the chronic stage, mineralized enthesophytes are present at the insertion, as well as irregular oblong calcifications [13]. Volume increase because of calcifications causes deformation of the Achilles tendon insertion, which results in painful swelling of the overlying subcutaneous tissue. Some patients with Haglund’s syndrome may have concurrent insertional and midportion tendinopathy.

Insertional tendinopathy in the absence of Haglund’s deformity is also possible due to overuse and mechanical factors. MRI reveals irregular proliferation of the bony attachment with adjacent alteration in the signals of tendons, enthesophytes and bursitis [18].

#### 2.2.1 MRI assessment of surgical treatment of Haglund's syndrome

**Fig. 14:**
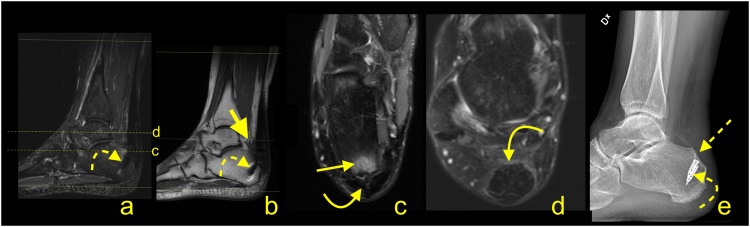
Normal status after the operation for Haglund's syndrome 9 months ago. Slight pain in the distal 1/3 of the Achilles tendon. MRI (a–d) and X-ray (e) revealed screws (curved dashed arrow) in the calcaneus and discrete postoperative bone marrow edema (straight arrow). The Achilles tendon is thick and fusiform but without rupture (curved arrow). Postoperative adhesions are visible in Kager's fat pad (thick arrow). A- T2- weighted with fat suppression, b- T1- weighted, c and d- PD- weighted with fat suppression, e- the lateral projection of the ankle joint.

**Fig. 15:**
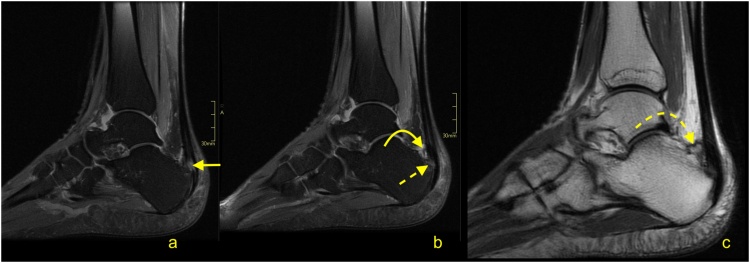
Status after the operation for Haglund's syndrome 6 months ago. Clinical improvement, reduction of pain, and swelling of Achilles tendon attachment. MRI revealed residual edema in the anterior part of the Achilles tendon insertion (straight arrow) with bone marrow edema in the calcaneus (dashed arrow). In the retrocalcaneal bursa, postoperative changes are visible as a slight inflammatory reaction (curved arrow) and adhesion (curved dashed arrow). A and b- PD- weighted with fat suppression, c- PD- weighted.

For patients with persistent symptoms who have not responded to nonoperative management, surgery is an option. MRI may confirm the clinical diagnosis and exclude problems that may produce similar manifestations (e.g., calcaneal stress fractures). The presence of the prominence on the posterosuperior part of the calcaneal tubercle may occur with or without inflammation in the adjacent structures. The presence of bone marrow edema within the bone deformation, retrocalcaneal bursitis and insertional tendinopathy comprise Haglund's syndrome and help in the selection of patients for surgery [17,19] (Fig. 13). The removal of the prominent posterosuperior process of the calcaneus is termed calcaneoplasty. This procedure is often accompanied by bursectomy, excision of pathological areas of the tendon and, if required, reattachment of the detached tendon (Fig. 14). Sometimes lengthening of the gastrocnemius aponeurotic recession, in the form of a V-Y plasty, is performed together with a flexor hallucis longus transfer [20,21]. These procedures can be performed endoscopically or under minimally invasive approaches [20,22]. Following surgery, the involved tissues show a high signal on MRI for a prolonged period of time, potentially in excess of a year (Fig. 15).

Complications following calcaneoplasty are uncommon and are going to be discussed briefly.

**Fig. 16:**
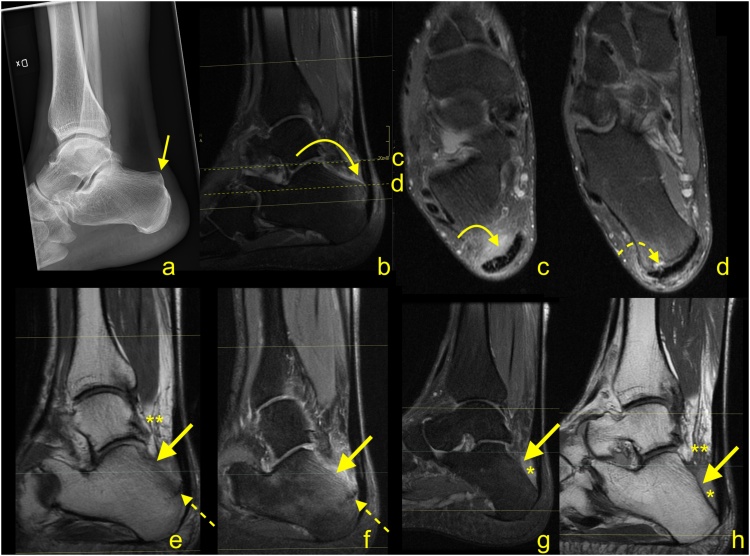
The postoperative complications for surgery to treat Haglund's syndrome. A 48-year-old patient presented with pain of the Achilles tendon insertion. X-ray (a) showed prominence of the supero-posterior part of the calcaneus (straight arrow). MRI (b, c and d) performed 3 weeks later revealed inflammation in the retrocalcaneal bursa, bone marrow edema in the bony prominence (curved arrow), and a cyst in the calcaneus (dashed curved arrow). In the postoperative period, the patient complained of pain in the heel, which was different than before the operation. The postoperative MRI (e, f, g and h) showed an incomplete fracture of the calcaneus (dashed arrow marks fracture line) near the Achilles tendon insertion and in the inferior part of the postoperative defect of the calcaneus (thick arrow). Postoperative reactive changes in Kager's fat pad (**). Postoperative fluid collection is visible between the calcaneus, Achilles tendon, and Kager's fat pad (*). A- the lateral projection of the ankle joint, b, c and d- PD- weighted with fat suppression, e- PD-weighted, f and g- PD- weighted with fat suppression, h- PD- weighted.

Iatrogenic calcaneal fracture (Fig. 16) may be seen as a result of the loss of volume. MRI reveals bone marrow edema in the posterior part of the calcaneus with a relatively distinct low intense line on T1-weighted images [17]. If contrast is administered, postoperative changes and bone marrow edema are going to show contrast enhancement.

**Fig. 17:**
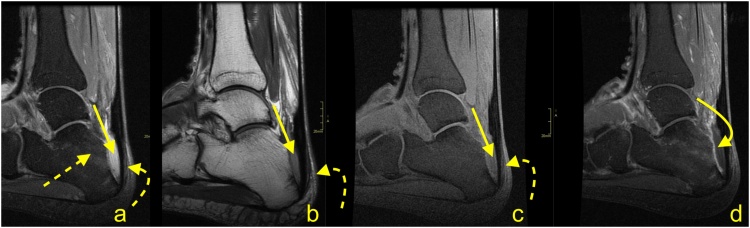
Fluid collection after surgery for Haglund's syndrome. Postoperative swollen heel (in an otherwise asymptomatic patient). MRI revealed a fluid collection (straight arrow) with discrete linear contrast wall enhancement (curved arrow), which indicates a seroma, and no evidence of abscess. Postoperative residual bone marrow edema (dashed arrow) in the calcaneus. Expected surgical thinning of the Achilles tendon is visible (curved dashed arrow). A- T2- weighted with fat suppression, b- PD- weighted, c-T1- weighted with fat suppression, d- T1- weighted with fat suppression and contrast.

A postoperative seroma (Fig. 17) is usually in the location where the calcaneus deformity has been removed. The contrast enhancement is discrete and seen only in the thin walls of the seroma. The seroma may be seen for a long time and most commonly does not cause any clinical symptoms [19,20].

**Fig. 18:**
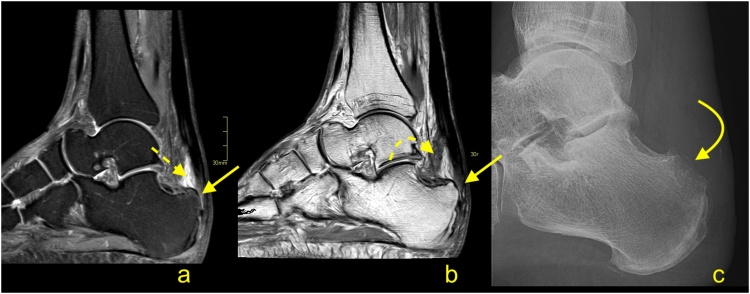
Chronic complication after surgery for Haglund’s syndrome. A 72-year-old patient presented 3 months after surgery for Haglund's syndrome with pain over the Achilles tendon insertion. The postoperative MRI (a and b) showed thinning of the Achilles tendon insertion (straight arrow), fluid in the retrocalcaneal bursa (curved arrow) and adhesions in Kager's fat pad (dashed curved arrow). Two months later, the patient presented with heel pain and reddening of the skin. Clinical suspicion of osteitis was confirmed on the X-ray (c), where osteolysis can be seen in the supero-posterior part of the calcaneus (curved arrow). A- T2- weighted with fat suppression, b- PD- weighted, c- the lateral projection of the calcaneus.

Infection (Fig. 18) may be a complication of any intervention, and it is usually a clinical diagnosis. Diagnostic imaging and bacteriological analysis are applied to confirm the diagnosis and to find the etiology [23]. Symptoms most often appear sometime after the procedure. First, they affect soft tissues, after some time, they can also affect the calcaneus. In the early stages, it is challenging to differentiate between infection and postoperative changes on MRI. Osteomyelitis of the calcaneus is seen as bone marrow edema with contrast enhancement [23]. If an abscess is present, a central fluid collection, destruction of bone and extensive contrast enhancement are visible. In more complicated cases, a fluid collection adjacent to the bone may be seen.

**Fig. 19:**
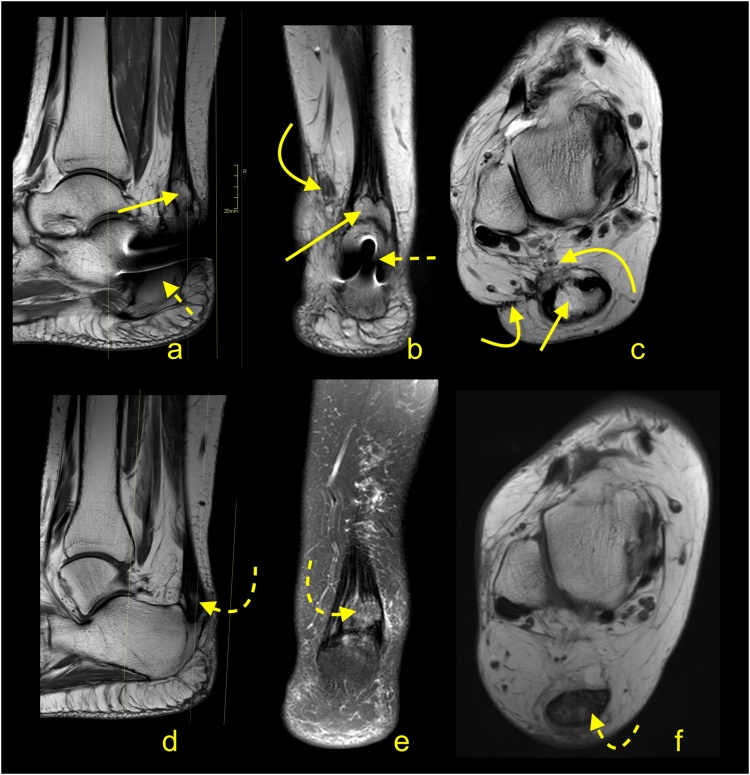
Recurrence of Haglund’s syndrome after surgery. A 71-year-old patient presented with pain and thickening of the Achilles tendon insertion about 1 year after surgery. Postoperative MRI (a–c) showed exostosis (straight arrow) in the Achilles tendon insertion, which is slightly bigger than the preoperative exostosis (curved dashed arrow) revealed on preoperative MRI (d-f). Fibrotic postoperative changes (curved arrow) are visible in Kager's fat pad. Screws in the calcaneus (dashed arrow). A- PD- weighted, b and c- T2- weighted, d- PD- weighted, e- T2- weighted with fat suppression, f- T1- weighted.

Recurrent Haglund’s deformity (Fig. 19) or adherences may be best visualized on MRI. Skin breakdown or partial contracture of the Achilles tendon may be best determined clinically [24].

## 3 Rupture of the Achilles tendon

Rupture of the Achilles tendon is a common sports injury [25]. Typical patients are male, over the age of 40 years and have recently returned to social sports that incorporate push offs [26]. Over the past three decades, there has been an increase in the frequency of Achilles tendon ruptures, which is estimated at 5–55 per 100,000 individuals [[27], [28], [29], [30], [31]]. The patient usually hears or appreciates a sudden "snap" in the calf or believes that they were hit from behind. Achilles tendon rupture may occur in the proximal part, in the midportion or in the distal part of the tendon. The most common site of a rupture is at the midportion of the tendon at 5–6 cm from the insertion, although ruptures may occur at the musculotendinous junction or close to the insertion on the calcaneus [32]. Two main theories concerning Achilles tendon rupture are that the rupture is due to degeneration of the tendon and the other is that novel stresses applied to an unconditioned tendon may cause a rupture [33].

### 3.1 Total ruptures

**Fig. 20:**
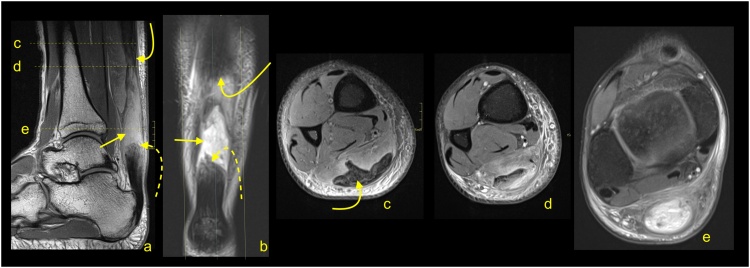
Total rupture of the midportion of the Achilles tendon (straight arrow), 3 weeks after trauma. MRI (a–e) showed that the proximal stump (curved arrow) and distal stump (dashed arrow) are frayed with significant diastasis. A and b- PD- weighted, c, d and e- PD- weighted with fat suppression.

**Fig. 21:**
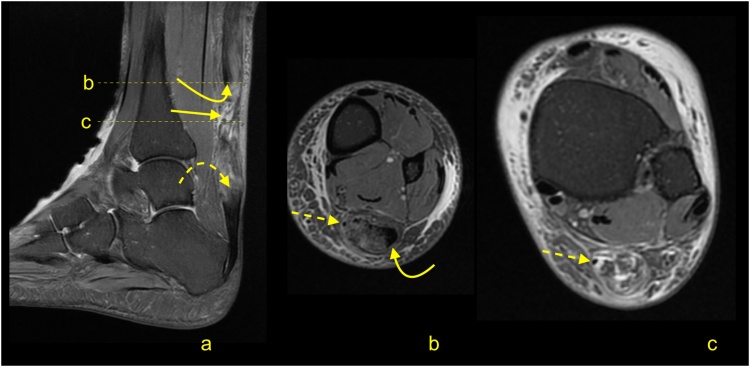
Achilles tendon rupture in midportion (straight arrow), 8 weeks after trauma. MRI (a–c) revealed fibrotic adhesions between the proximal stump (curved arrow) and distal stump (dashed arrow). The plantaris tendon is preserved (dashed arrow). A, b and c- PD- weighted with fat suppression.

**Fig. 22:**
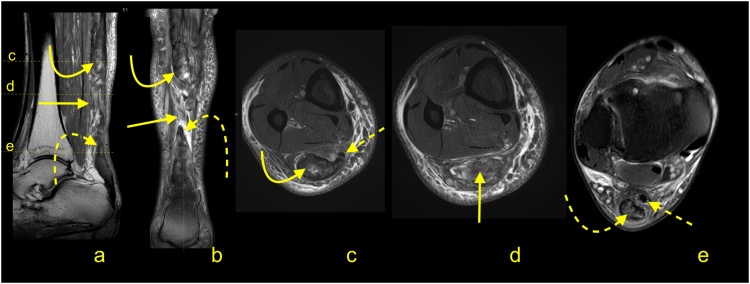
Achilles tendon rupture in midportion (straight arrow). MRI performed 4 months after the trauma (a-e). Fibrotic adhesions fill in the space between the proximal stump (curved arrow) and distal stump (dashed curved arrow). Outlines of the stumps are regular. The plantaris tendon is ruptured (dashed arrow). A- PD- weighted, b- T2- weighted with fat suppression, c, d and e- PD- weighted with fat suppression.

**Fig. 23:**
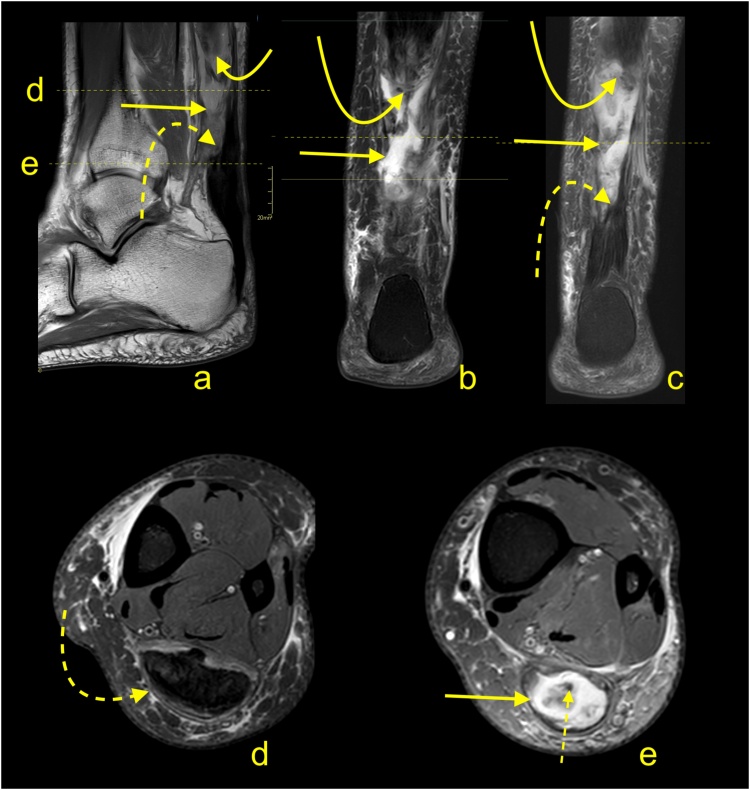
Achilles tendon rupture in midportion (straight arrow). MRI preformed 9 months after the trauma (a–e). No adhesions are visible between the proximal stump (curved arrow) and distal stump (dashed curved arrow). The paratenon at the level of rupture is filled with fluid (straight arrow). The plantaris tendon is preserved (dashed straight arrow). A- PD- weighted, b and c- T2- weighted with fat suppression, d and e- PD- weighted with fat suppression.

**Fig. 24:**
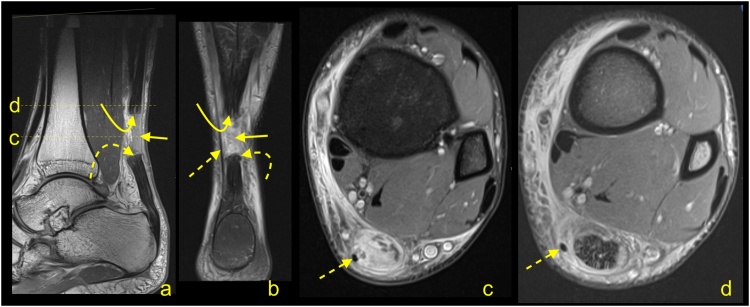
Achilles tendon rupture in midportion (straight arrow). A 36-year-old patient receiving systemic corticosteroid therapy. MRI (a–d) revealed that no adhesions are visible between the proximal stump (curved arrow) and distal stump (dashed curved arrow). Very regular outlines of stumps are visible. The plantaris tendon is preserved (dashed arrow). A- PD- weighted, b- T2- weighted with fat suppression, c and d- PD- weighted with fat suppression.

Total ruptures occur most commonly at the midportion of the tendon, and the ends of the tendon are frayed with a gap between the tendon ends (Fig. 20, Fig. 21, Fig. 22, Fig. 23). On MRI, the slice thickness together with the position of the tendon in the scanner contribute to challenges in determining the actual distance that tendon ends are separated. This may be most apparent on sagittal sections, however, assessing the coronal section is also helpful (Fig. 23). We observed less fraying or almost regular edges when rupture was a consequence of systemic steroid therapy (Fig. 24).

**Fig. 25:**
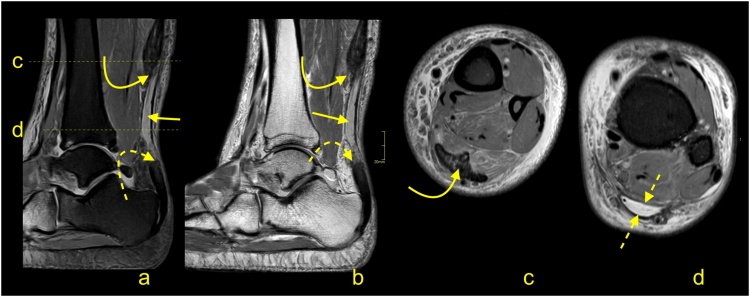
Chronic Achilles tendon rupture in midportion (straight arrow). A 46-year-old patient who suffered a ruptured Achilles tendon 2 years ago. MRI (a–d) revealed that the paratenon space is filled by fluid, and adhesions are visible between the proximal stump (curved arrow) and distal stump (dashed curved arrow). The paratenon is not distended, which indicates its chronic character (dashed arrow). A- T2- weighted with fat suppression, b- PD- weighted, c and d- PD- weighted with fat suppression.

Significant diastasis between the stumps of the tendon may be an indication for surgery [34,35]. MRI may also reveal concurrent muscle tears or contusions from direct blows (Fig. 20). The total Achilles tendon injury may be associated with paratenon tear and plantaris tendon rupture (Fig. 22). In the acute phase after injury, the paratenon and space between the tendon stumps are filled by blood (Fig. 21). With significant trauma, the hematoma may extend anteriorly into Kager's fat pad. Over the first few days following injury, the hematoma is resorbed making the outline of the paratenon more sunken (Fig. 25). With further time and inflammation following injury, the frayed tendon stamps become more regular, and the separation between the tendon stumps becomes more apparent (Fig. 23). A somewhat focal protrusion of Kager's fat pat can be observed at the level of injury. If the tendon does not heal, an interposed pseudotendon forms; however, it cannot transmit forces between the muscle and the bony insertion. This may cause the gastrocnemius and soleus muscles to develop fat atrophy. These cases may be amenable to reconstruction using a gastrocnemius turndown, free flap or a tendon transfer through transosseous calcaneal tunnels. A comparison of differences in tendon length is useful for planning surgery; however, this requires MRI images of both legs to be obtained, requiring additional scanning time and can be easily evaluated on ultrasound.

**Fig. 26:**
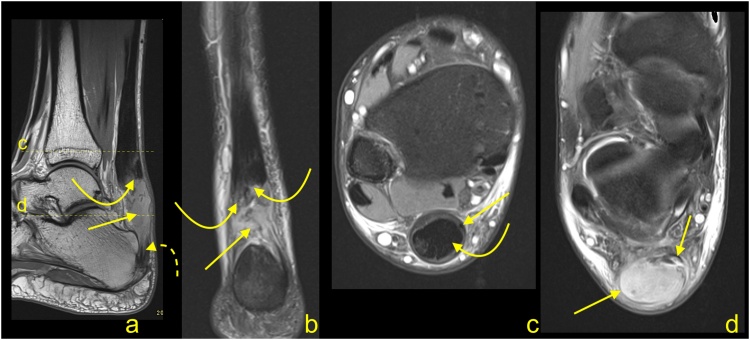
Total Achilles tendon rupture in the distal part 3 weeks ago. MRI showed that asymmetry of stumps is visible. The proximal stump is larger (curved arrow) while the distal stump is smaller (dashed curved arrow). The space between the stumps is filled with fluid (straight arrow). The plantaris tendon is preserved and contributes to the paratenon (dashed arrow). A- PD- weighted, b- T2- weighted with fat suppression, c and d- PD- weighted with fat suppression.

**Fig. 27:**
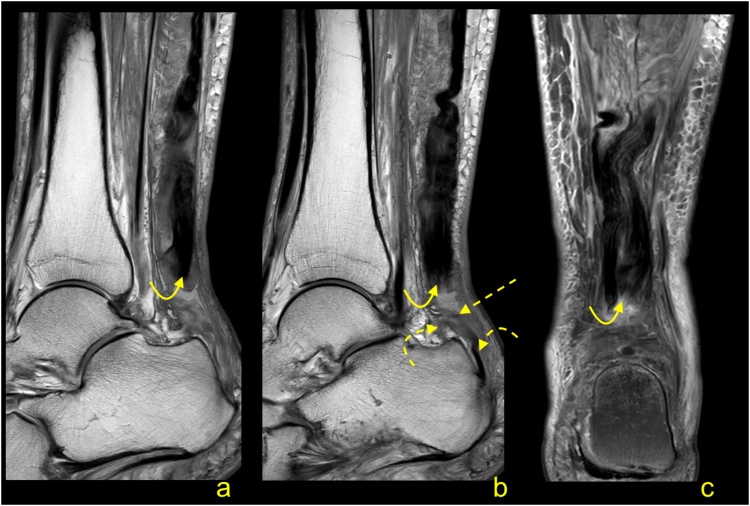
Total Achilles tendon rupture in the distal part 1 week ago (dashed arrow). MRI revealed that the proximal stump had retraction and shortening (curved arrow). The distal stump is frayed with volume reduction (curved dashed arrow). A and b- PD- weighted, c- T2- weighted with fat suppression.

**Fig. 28:**
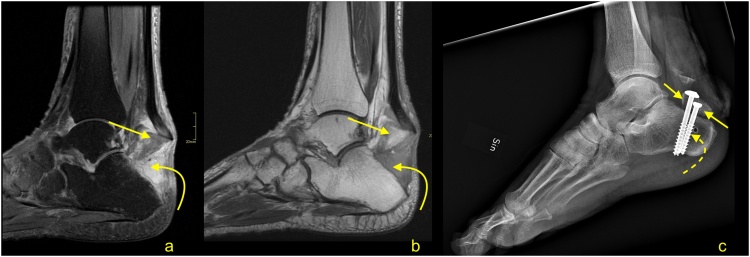
Comminuted avulsion fracture of the calcaneus. Car accident. Large bone fragment (straight arrow) with the attachment of Achilles tendon detached from the calcaneus (a and b- MRI). At the level of the fracture hematoma (curved arrow). X-ray (c) showed that the largest bone fragments are fixed to the calcaneus (dashed curved arrow). The incomplete postoperative function of the Achilles tendon. A- T2- weighted with fat suppression, b- PD- weighted, c- the lateral projection of the ankle joint.

The distal total rupture of the Achilles tendon within 2 cm of the insertion is fortunately rare [36]. The treatment of distal ruptures is much more challenging than midportion ruptures due to minimal amounts of distal soft tissues and the likelihood of pre-existing tendinopathy [37] (Fig. 25, Fig. 26, Fig. 27). MRI reveals the fluid in the paratenon, fraying of the stumps, residual distal tendon stumps, and a thick paratenon (Fig. 26). Bone marrow edema together with edema in Kager's fat pad may also be present. The proximal stump may be wrinkled (Fig. 27), while diastasis is not usually as prominent (Fig. 26, Fig. 27), as for midsubstance ruptures. In rare cases, a distal Achilles tendon injury may include a bony avulsion, often with significant diastasis because of muscle activity (Fig. 28). Avulsed bony fragments usually require surgical reattachment to the calcaneus.

**Fig. 29:**
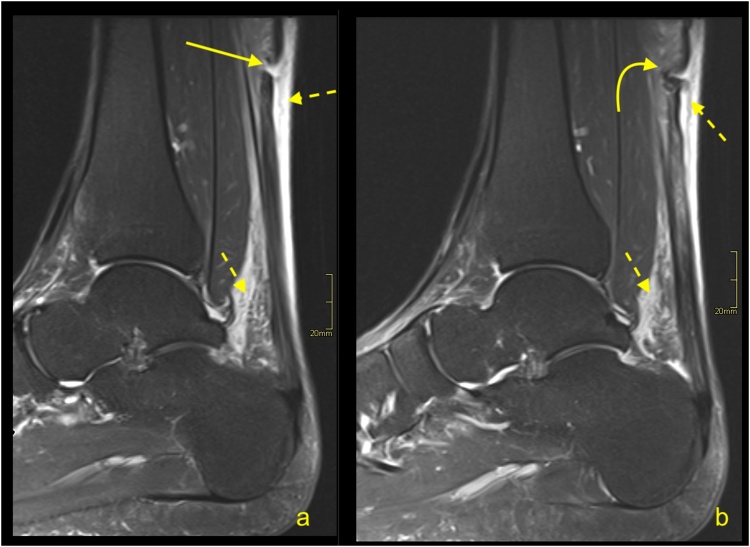
Proximal Achilles tendon rupture. A 32-year-old athlete played squash and felt a sudden pain in the upper part of the Achilles tendon. The Achilles tendon defect is visible (straight arrow) on MRI (a and b); the remaining part of the tendon is wavy-shaped (curved arrow). Reactive changes in the subcutaneous tissue and Kager’s fat pad (dashed arrow). A and b- PD- weighted with fat suppression.

**Fig. 30:**
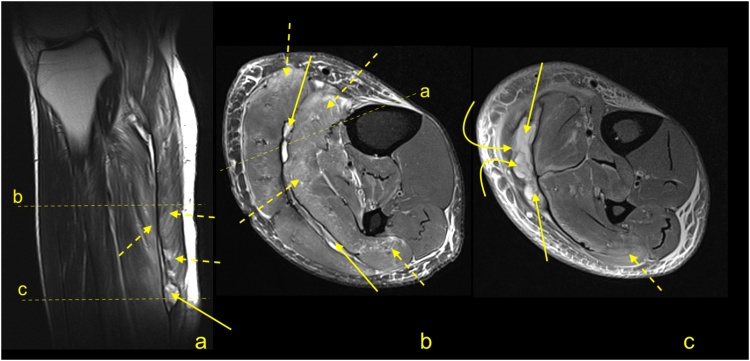
A 25-year-old man who played football and at the end of training felt acute pain in the calf. MRI (a–c) showed a hematoma separating the aponeuroses of the gastrocnemius and soleus muscles (straight arrow) and extensive muscle edema in the soleus and gastrocnemius muscles (dashed arrow). The defect at the musculotendinous junction of the medial head of the gastrocnemius is visible (curved arrow). A- PD- weighted, b and c- PD- weighted with fat suppression.

Proximal ruptures of the Achilles tendon are rarer than midsubstance tears and are more likely to be partial tears (Fig. 29). They often occur in younger patients and are related to engagement in sports activity without a proper warm-up [38]. MRI features are like those seen in other parts of the tendon and include fraying of the tendon and hematoma accumulation. Less retraction of the proximal stump occurs because they are more likely to be partial ruptures and tears extending into the myotendinous junction (Fig. 30), so surgery offers a less clear benefit if performed.

### 3.2 Partial ruptures

Partial ruptures of the Achilles tendon may occur in any part of the tendon and may be located at the surface or intratendinous. The differential diagnosis of proximal Achilles tendon ruptures includes tears of the myotendinous junction and gastrocnemius muscle contusion (Fig. 30). Most partial surface tears may be related to tendon degeneration. Most of them have a vertical orientation and are located in the dorsal part of the tendon, affecting the subtendon from the medial head of the gastrocnemius [[39], [40], [41], [42]]. Intratendinous defects are located mainly in the anteromedial part of the tendon, affecting mostly the subtendons from the lateral caput of the gastrocnemius and soleus muscles. Partial intratendinous ruptures are seen in thickened tendons related to tendinopathy, which is most commonly seen in the midportion of the tendon. Partial tears secondary to Haglund’s syndrome are located distally and anteriorly.

#### 3.2.1 MRI assessment after reconstruction and conservative treatment of the Achilles tendon

**Fig. 31:**
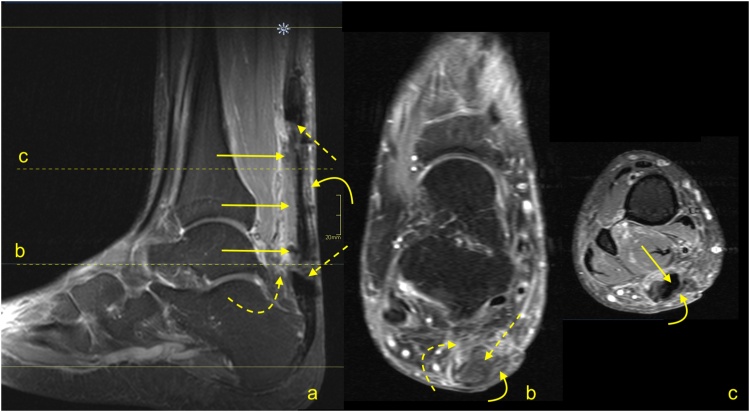
Status after Achilles tendon reconstruction 2 years ago. Asymptomatic patient, with good clinical function of the tendon. MRI (a–c) revealed the preserved tendon continuity with the native tendon (dashed arrow). The reconstructed tendon segment has a heterogeneous signal and an irregular outline (straight arrow), which are expected postoperative changes. A slightly higher signal of Kager's fat pad (curved dashed arrow) and the paratenon (curved arrow) are postoperative changes. A- T2- weighted with fat suppression, b and c- PD- weighted with fat suppression.

Following repair and/or reconstruction, the tendon or graft incorporates, and the resultant healing tendon becomes irregular and thicker. If sutures were used, the continuity of surgical threads should be checked. In case of the presence of an osseous canal, T1-weighted images may be relevant to assess tunnel position and to exclude a fracture. Graft healing is a complex and long process; thus, the signal is usually heterogeneous and of greater intensity over potentially several years. Interpretation of the higher Achilles tendon signal after surgery should be related to the clinical findings (Fig. 31) [33,43].

One of the most important indications for MRI of the Achilles tendon is for its assessment following suspected re-injury and other management of complications. Re-ruptures occur in about 2–5 % of patients, most often 4–13 weeks after repair or the commencement of nonsurgical management [44]. Re-rupture of the sutured tendon may be seen on MRI as fluid located beneath a thickened paratenon. Often fraying and retraction of the healing fibers are visible, and the paratenon is usually thickened because of postoperative changes.

**Fig. 32:**
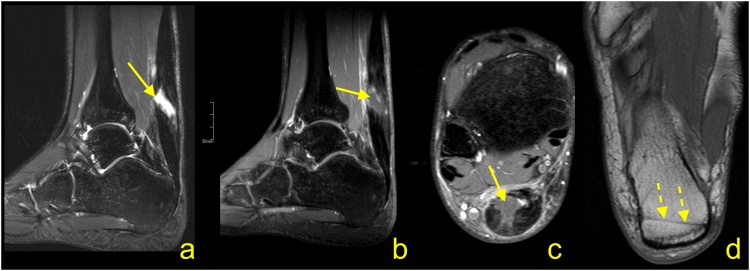
Status after Achilles tendon reconstruction 14 months ago. Patient presenting with pain in the midportion of the Achilles tendon. The skin over this area is somewhat changed, and reddening is visible. Clinical suspicion of infection. MRI with contrast was performed (a–d). At the level of the sutures, there is a visible substance defect in the tendon, which is oriented spirally (straight arrow) and filled with fluid (a). Only linear contrast enhancement is seen as no evidence of an abscess (b and c). Reinforcement sutures passing through the osseous tunnel in the calcaneus is seen on T1-weighted imaging (d - dashed arrow). a – T2-weighted with fat suppression, b and c- T1-weighted with fat suppression and iv contrast, d- T1- weighted.

**Fig. 33:**
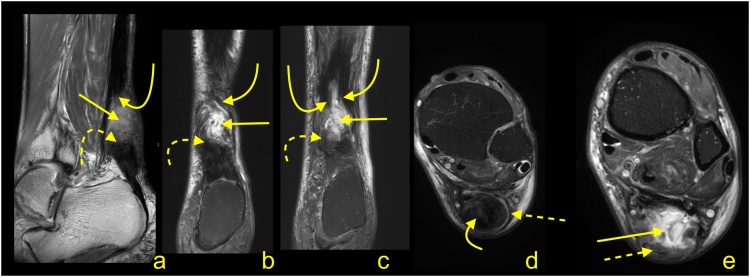
Status after conservative treatment of the Achilles tendon rupture about 4 months ago. MRI revealed that no adhesions are visible between the proximal stump (curved arrow) and distal stump (dashed curved arrow). Very irregular outlines of stumps are noticed. The paratenon is preserved but thick (dashed arrow). A- PD- weighted, b and c- T2- weighted with fat suppression, d and e- PD- weighted with fat suppression.

A discontinuity and unambiguously fluid signal within the postoperative tendon (Fig. 31, Fig. 32) or after conservative treatment indicate rupture (Fig. 33). A properly functioning tendon should not be wavy or relaxed in the position of 90 grades plantar flexion. From experience, the MRI examination with the ankle in a neutral position is better to assess the tension of the reconstructed tendon. When more plantar flexion is applied, the tendon is more relaxed, which makes it more challenging to assess the tension. Hence, the evaluation of the Achilles tendon in the routine ankle protocol may be insufficient. If postoperative infection is clinically suspected, contrast administration is recommended.

**Fig. 34:**
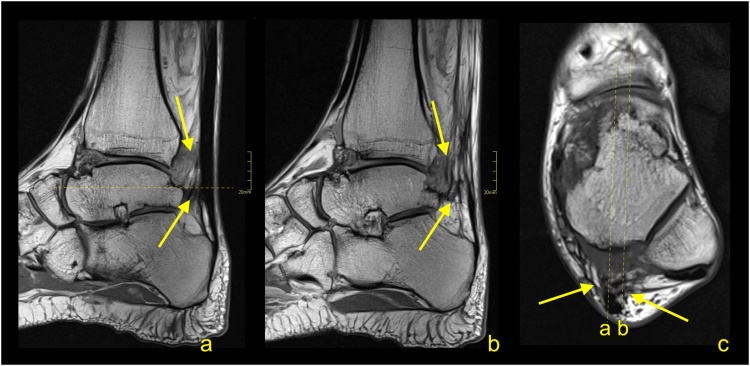
Status after elongation of the Achilles tendon in childhood. Clinical stiffness and reduced mobility in the ankle joint. MRI (a–c) revealed the extensive postoperative changes between the Achilles tendon and Kager's fat pad (straight arrow). A, b and c- PD- weighted.

The most common postoperative lesions are adhesions, which are located between the Achilles tendon, paratenon, and adjacent structures, most common in Kager's fat pad (Fig. 34). Adhesions may at times cause stiffness and reduced mobility. In this instance, dynamic ultrasonography is superior to MRI.

## 4 Tendon elongation

**Fig. 35:**
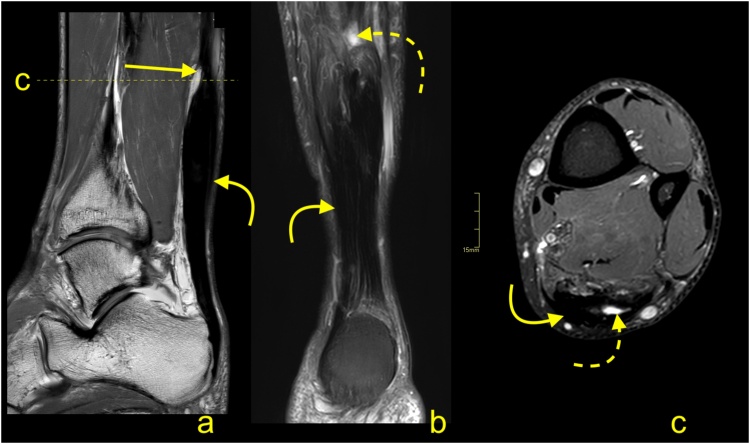
Chronic Achilles tendon rupture with some dynamic inefficient. A 58-year-old patient who suffered a ruptured Achilles tendon 1 year ago. Clinical suspicion of tendon elongation. The MRI (a–c) revealed a thickened and relaxed Achilles tendon, which is a sign of elongation. In the proximal part of the tendon, a posttraumatic substance defect in the tendon is visible (straight arrow), and in the midportion, thickening of the tendon is present (curved arrow). In the defect, a small amount of fluid is visible (dashed curved arrow). A- PD- weighted, b- T2- weighted with fat suppression, c- PD- weighted with fat suppression.

The elongation of the Achilles tendon after surgical repair or conservative treatment may influence the clinical outcome and may cause a reduction of plantar flexion of the ankle [45,46]. Elongation occurs mostly in 6–12 weeks after repair, which requires earlier prevention [47,48]. The degree of elongation is variable and is approximately 5–11 mm in the first 6–7 weeks, and if it progresses, it can reach 8–14 mm after 3 months [45,46,49]. The degree of tendon elongation is clinically relevant and should be provided in the radiological report [45,50]. Comparison with the healthy side or with previous examinations, if available, is beneficial. An elongated tendon has a wavy course and a heterogeneous structure due to previous tear and remodeling [50] (Fig. 35).

## 5 Summary

Clinical assessment of painful Achilles tendon is often sufficient. However, diagnostic imaging may play a decisive role in qualifying for treatment. In the case of Achilles tendon injuries, the role of diagnostic imaging is to determine the degree of injury. It is essential to decide on the diastasis between the tendon stumps in total ruptures because this may determine surgical treatment. Assessing the degree of tendon elongation is necessary for planning surgery. Paratendinitis should be differentiated from midportion tendinopathy because treatment of tendinopathy is somewhat different. The important role diagnostic imaging plays in the early detection of tendinopathy and possible coexisting partial tear of the Achilles tendon. Following surgery, there is a higher signal of the Achilles tendon. The fluid signal within the Achilles tendon graft indicates a rupture. The abnormalities on MRI should be correlated with the clinical findings.

## Declaration

The Swedish Ethics Committee approved the study and waived the need for informed consent (2020−06177). This project did not receive any specific grant from funding agencies in the public, commercial, or not-for-profit sectors. The authors declare that there is no conflict of interest.

## Author’s contribution

Conceptualization, Investigation, Visualization, Supervision, Roles/Writing - original draft: PS. Investigation, Writing - review & editing: PS, KNH, MC. All authors accepted the final version of the manuscript.

## Founding

This project did not receive any specific grant from funding agencies in the public, commercial, or not-for-profit sectors.

## Declaration of Competing Interest

The authors report no declarations of interest.
